# MRI Assessment of Changes in Tumor Vascularization during Neoadjuvant Anti-Angiogenic Treatment in Locally Advanced Breast Cancer Patients

**DOI:** 10.3390/cancers15184662

**Published:** 2023-09-21

**Authors:** Torgeir Mo, Siri Helene Bertelsen Brandal, Oliver Marcel Geier, Olav Engebråten, Line Brennhaug Nilsen, Vessela N. Kristensen, Knut Håkon Hole, Tord Hompland, Thomas Fleischer, Therese Seierstad

**Affiliations:** 1 Faculty of Clinical Medicine, University of Oslo, 0316 Oslo, Norway; torgeir.mo@medisin.uio.no (T.M.); sibert@ous-hf.no (S.H.B.B.); olav.engebraten@medisin.uio.no (O.E.); v.n.kristensen@medisin.uio.no (V.N.K.); khh@ous-hf.no (K.H.H.); 2 Department of Cancer Genetics, Institute for Cancer Research, Oslo University Hospital, 4950 Oslo, Norway; thomas.fleischer@rr-research.no; 3 Department of Breast Diagnostic, Oslo University Hospital, 0379 Oslo, Norway; 4 Department of Diagnostic Physics, Oslo University Hospital, 0379 Oslo, Norway; ogeier@ous-hf.no; 5 Department of Oncology, Oslo University Hospital, 0379 Oslo, Norway; 6 Department of Tumor Biology, Institute for Cancer Research, Oslo University Hospital, 4950 Oslo, Norway; 7 Department of Medical Physics, Oslo University Hospital, 0379 Oslo, Norway; linen@ous-hf.no; 8 Department of Medical Genetics, Oslo University Hospital, 0450 Oslo, Norway; 9 Department of Oncologic Radiology, Division of Radiology and Nuclear Medicine, Oslo University Hospital, 0379 Oslo, Norway; 10 Department of Radiation Biology, Oslo University Hospital, 4950 Oslo, Norway; tord.hompland@rr-research.no; 11 Department of Research and Development, Division for Radiology and Nuclear Medicine, Oslo University Hospital, 0379 Oslo, Norway

**Keywords:** breast neoplasms, magnetic resonance imaging, angiogenesis inhibitors, pharmacokinetics, neoadjuvant therapy, fractals

## Abstract

**Simple Summary:**

Experimental and clinical studies have revealed that vascular endothelial growth factor (VEGF) is the predominant angiogenic factor in breast cancer. VEGF expression correlates with inferior outcomes and advanced-stage breast cancer. Bevacizumab is a humanized anti-VEGF monoclonal antibody and has been shown to improve response rates in the treatment of breast cancer, but has failed to improve progression-free survival or overall survival. Anti-VEGF treatment can temporarily normalize tumor vascularization and it is hypothesized that there might be a window of opportunity for chemotherapy. Dynamic contrast-enhanced MRI (DCE-MRI) is the most accurate radiological tool to aid in staging and treatment monitoring of advanced breast cancer. In this work, we showed that DCE-MRI is a sensitive tool to measure the treatment effect of bevacizumab and that it shuts down the vascularization early and abruptly. DCE-MRI may be a suitable tool to find an eventual therapeutic window, and possibly identify a subgroup that would benefit the most from anti-VEGF treatment.

**Abstract:**

Anti-VEGF (vascular endothelial growth factor) treatment improves response rates, but not progression-free or overall survival in advanced breast cancer. It has been suggested that subgroups of patients may benefit from this treatment; however, the effects of adding anti-VEGF treatment to a standard chemotherapy regimen in breast cancer patients are not well studied. Understanding the effects of the anti-vascular treatment on tumor vasculature may provide a selection of patients that can benefit. The aim of this study was to study the vascular effect of bevacizumab using clinical dynamic contrast-enhanced MRI (DCE-MRI). A total of 70 women were randomized to receive either chemotherapy alone or chemotherapy with bevacizumab for 25 weeks. DCE-MRI was performed at baseline and at 12 and 25 weeks, and in addition 25 of 70 patients agreed to participate in an early MRI after one week. Voxel-wise pharmacokinetic analysis was performed using semi-quantitative methods and the extended Tofts model. Vascular architecture was assessed by calculating the fractal dimension of the contrast-enhanced images. Changes during treatment were compared with baseline and between the treatment groups. There was no significant difference in tumor volume at any point; however, DCE-MRI parameters revealed differences in vascular function and vessel architecture. Adding bevacizumab to chemotherapy led to a pronounced reduction in vascular DCE-MRI parameters, indicating decreased vascularity. At 12 and 25 weeks, the difference between the treatment groups is severely reduced.

## 1. Introduction

Vascular endothelial growth factor (VEGF) is a predominant angiogenic factor in breast cancer. VEGF expression correlates with advanced-stage breast cancer and inferior outcomes [1]. The American Food and Drug Administration (FDA) approved the use of the anti-angiogenic inhibitor bevacizumab for the treatment of metastatic human epidermal growth factor receptor 2 (HER2)-negative breast cancer in 2008. The lack of improvement in overall survival combined with more adverse effects ultimately resulted in the FDA revoking the approval of bevacizumab as a treatment for breast cancer in 2011 [2].

Even though many clinical breast cancer studies point to some increase in progression-free survival, none could show statistically significant improvement in overall survival for patients treated with VEGF suppressors [3]. The effect is inconsistent [4], but it has been hypothesized that a small subset of breast cancer patients may benefit from VEGF-suppressing treatment [5]. In our NeoAva clinical trial, patients were randomized 1:1 to receive bevacizumab + chemotherapy or chemotherapy alone, and we observed a larger occurrence of pathological complete response (pCR) in the combination arm for ER-positive patients [6]. More recently, we have also shown that protein expression and DNA methylation can define subgroups of patients that benefit from the treatment combination [7].

The effect on vascular function and architecture, and the resulting effect on blood, oxygen, and drug distribution of adding bevacizumab to standard chemotherapy is uncertain, but based on its mechanism of action, aiming at disrupting the angiogenic process, it was originally anticipated that it would reduce blood perfusion by shutting down the vasculature. Contrary hypotheses have been proposed, however, suggesting that the disruption of the angiogenic process results in increased blood perfusion through a normalization of the vasculature, making it more efficient [8,9].

Non-invasive in vivo dynamic contrast-enhanced magnetic resonance imaging (MRI) is a routine clinical tool for longitudinal assessment of changes in tumor vascularization. Semi-quantitative measures or pharmacokinetic modeling of the exchange of contrast agent between the vasculature and the extravascular-extracellular space can provide information on the functionality of the tumor vasculature, whereas texture analysis, in particular the fractal geometry of the image texture of the DCE-MRI images, can provide information on the tumor vascular architecture [10].

The aim of this study was to investigate the effects of anti-angiogenic treatment on tumor vascular functionality and vascular architecture as assessed by DCE-MRI. We compared longitudinal DCE-MRI data between patients receiving chemotherapy or chemotherapy + bevacizumab.

## 2. Materials and Methods

### 2.1. Patient Cohort

The study cohort consisted of 70 patients with large (≥2.5 cm) HER2-negative breast tumors (Table 1). Patients were recruited between November 2008 and July 2012 at Oslo University Hospital as part of a larger multicenter study (NeoAva [6]; Clinical Trials ID NCT00773695). All patients provided written informed consent prior to inclusion. The institutional protocol review board, the regional ethics committee, and the Norwegian Medicines Agency approved the study, and the study was carried out in accordance with the Declaration of Helsinki, International Conference on Harmony/Good Clinical Practice.

### 2.2. Study Protocol

Patients received neoadjuvant chemotherapy consisting of four cycles of FEC100 (5 flourouracil 600 mg/m^2^, cyclophosphamide 600 mg/m^2^, and epirubicin 100 mg/m^2^) every three weeks, followed by either four cycles of 100 mg/m^2^ docetaxel every three weeks, or 12 weekly infusions of paclitaxel at a dose of 80 mg/m^2^. Thirty-eight randomly selected patients were given additional intravenous administration of the VEGF inhibitor bevacizumab at a dose of 10 mg/kg every other week, or 15 mg/kg every third week in patients receiving paclitaxel or docetaxel, respectively.

### 2.3. MRI Examinations

MRI examinations were performed at four different timepoints: before treatment (*n* = 70), after 1 week (*n* = 26), after 12 weeks (*n* = 67), and after 25 weeks of treatment (*n* = 65). Surgery was performed within two weeks after the last MRI exam. The MR exams were performed using a 1.5 T MR scanner (Siemens Magnetom ESPREE, Siemens, Erlangen, Germany) and an 8-channel breast coil (CP breast coil, Siemens, Erlangen, Germany). The MRI protocol was designed in accordance with the EUSOBI recommendations [11] Mann 2019 and included T2-weighted, diffusion-weighted and dynamic contrast-enhanced (DCE) MRI. The DCE-MRI was performed using a 3D spoiled gradient echo (SPGR) with radial sampling and k-space weighted (T1) image contrast [12,13] (TE = 2.56 ms, TR = 5.46 ms, spatial resolution = 1.0 mm × 1.0 mm × 1.5 mm, temporal resolution 13 s, spectral adiabatic inversion recovery fat suppression) (Supplementary Table S1). Contrast agent was distributed at an injection flow rate of 3 mL/s, at a dose of 0.08 mmol/kg body weight, using the gadolinium-based contrast agent Gadovist (Bayer Pharma AG, Leverkusen, Germany). Two sets of T1-weighted SPGR images with identical slice positioning as the DCE images were also acquired, one using the breast coil, and one using the body coil. These acquisitions were used to correct for the inhomogeneous B1-profile of the breast coil. The study was conducted according to the recommendations for appropriate methodology for clinical trials for assessment of anti-angiogenic and anti-vascular therapeutics using MRI [14,15]. 

### 2.4. Tumor Volumetry

Two separate radiologists prospectively interpreted the clinical MRI examinations and identified the tumor extent. Based on this, a volume was defined on the axial subtraction images that covered the whole extent of the tumor. A marching-squares algorithm with an iso-level set by a local Otsu threshold was used in this volume to automatically delineate the tumor(s). Next, a third experienced radiologist (SHBB) adjusted the automatically segmented tumor volumes using all sequences from the multiparametric examination (Supplementary Table S1). The radiologist excluded necrotic regions identified through the combination of high apparent diffusion coefficient (ADC) and no contrast uptake on DCE.

Tumor volume (V) was obtained by multiplying the number of pixels within the volume of interest (VOI) with the pixel spacing and the slice thickness. 

### 2.5. Tumor Perfusion

Motion correction was applied in patients showing bulk motion during the DCE imaging, using commercially available software [16]. B1-inhomogeneities were corrected by multiplying the motion-corrected DCE images with the ratio between the T1-weighted images acquired using the breast coil and the T1-weighted images obtained using only the body coil.

The semi-quantitative analysis consisted of calculation of time-to-peak (TTP, time at which the contrast concentration reaches its maximum) and the area under the contrast curve (AUC) during the first 90 s of contrast enhancement for all voxels within the tumor.

The quantitative analysis was performed using the extended Tofts model (ETM) [17,18]. The extended Tofts model is a special variant of a two-compartment exchange model where the contrast-enhancing molecular tracer is assumed to occupy either the vascular or the extravascular–extracellular tissue space, with relative volumes $v_{p}$ and $v_{e}$, respectively. The dynamics of this system are governed by the rate at which tracer diffuses between the two compartments ($K^{trans}$). The distribution of molecular contrast enhancing tracer over time is regarded as a response to an arterial input function (AIF), describing the concentration of tracer in the feeding artery:

(1) $$C_{t}\left(t\right)=K^{trans}\exp\left(-k_{ep}t\right)*C_{p}\left(t\right)$$

where $C_{t}\left(t\right)$ is the total tissue concentration of tracer (i.e., the total image contrast enhancement), $C_{p}\left(t\right)$ is the plasma concentration (AIF). $K^{trans}/v_{e}=k_{ep}$ is the standard notation. ∗ denotes the convolution operation. 

A population AIF was created by sampling the signal from the left ventricle on B1-normalized pretreatment image series from 20 patients selected randomly from both treatment cohorts. Using a population AIF rather than an individually sampled AIF has been shown to increase reproducibility in cases where the SNR is limited, or where minor artifacts resulting from B1-normalization can hamper robust AIF sampling in individual patients [19]. The extended Tofts model was implemented using nICE (Nordic NeuroLab, Bergen, Norway), where fixed pre-contrast T1-relaxation times were set to 1200 ms and 900 ms for blood and breast tissue, respectively [20,21,22]. Parametric maps of $K^{trans},k_{ep},v_{p},v_{e}$ were created for all tumors at all examination timepoints. 

### 2.6. Tumor Vascular Architecture—Texture Analyses

Tumor vasculature has a more chaotic structure than normal vasculature. Baish and Jain introduced a framework for using fractal analysis to measure the complexity of the vascular architecture and demonstrated that the fractal dimensionality of tumor vessel growth patterns is higher than in normal vessels [10].

Michallek et al. found that the vascular tortuosity was strongly correlated to image texture, specifically to the local fractal dimension of the post-injection subtraction images in contrast-enhanced MRI of prostate cancer patients [23]. Building on this work, the local fractal dimensionality of the tumor images in this study was calculated. Subtraction images were created by subtracting the mean of five pre-injection images from the 238 s post-injection image. The time of 238 s post-injection was chosen because it was the closest value to the median TTP value in all the pretreatment tumors. In the subtraction images, fractal dimension was calculated in a sliding window consisting of 3 × 3 voxels, using the blanket method [24], as per the recommendations adhered to by Michallek et al. The number of blankets used was 44, corresponding to the value that gives the closest equality of fractal dimension estimate between a 3 × 3 window, and a 255 × 255 window of a self-similar Brodatz texture image (D04) [24], and is the same as reported by Michallek et al. [23]. For each tumor, a representative fractal dimension value was calculated from the median value of tumor voxels situated at minimum one voxel away from the border towards normal tissue.

### 2.7. Statistics

The main metrics analyzed are the relative, or percentage change in the median value of the respective parameters for each tumor. The change, at any time point, is measured relative to the pre-treatment value. Unless otherwise specified, statistical *p*-values are calculated using a two-sided Mann–Whitney test. The statistical significance level is defined as *p* < 0.05.

To minimize data loss, pairwise deletion was used for handling missing values between different analyses. Assuming that the missing data are relatively randomly distributed between cases, the risk of data bias is low.

Because the tumors undergo significant volume reduction during treatment, distributions are shown as probability densities rather than frequency histograms. A probability density plot reflects the frequency at which a value appears in the sample and is thus similar to a histogram normalized to the sample size. 

## 3. Results

### 3.1. Tumor Volumetry

**Figure 1:**
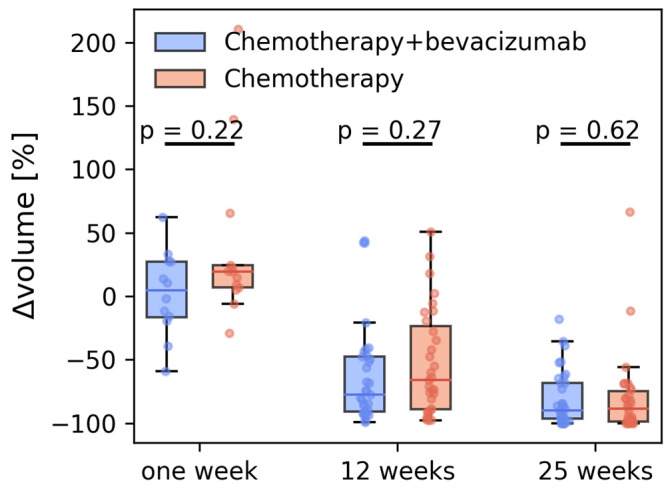
Relative change in MRI-derived tumor volumes after 1, 12, and 25 weeks for the two treatment groups.

At baseline, the mean tumor volume for the study cohort was 10.45 cm^3^ (1.8–113.7 cm^3^) and there was no difference between the two treatment groups (*p* = 0.19). There was no difference in tumor volume or tumor volume change between the two treatment groups at any of the four timepoints (Table 2, Figure 1). At 1 week there was no significant change in tumor volume; however, at 12 weeks and 25 weeks large significant reductions in mean tumor volume, 73.7% and 89.8%, respectively, were observed.

### 3.2. Tumor Perfusion

**Figure 2:**
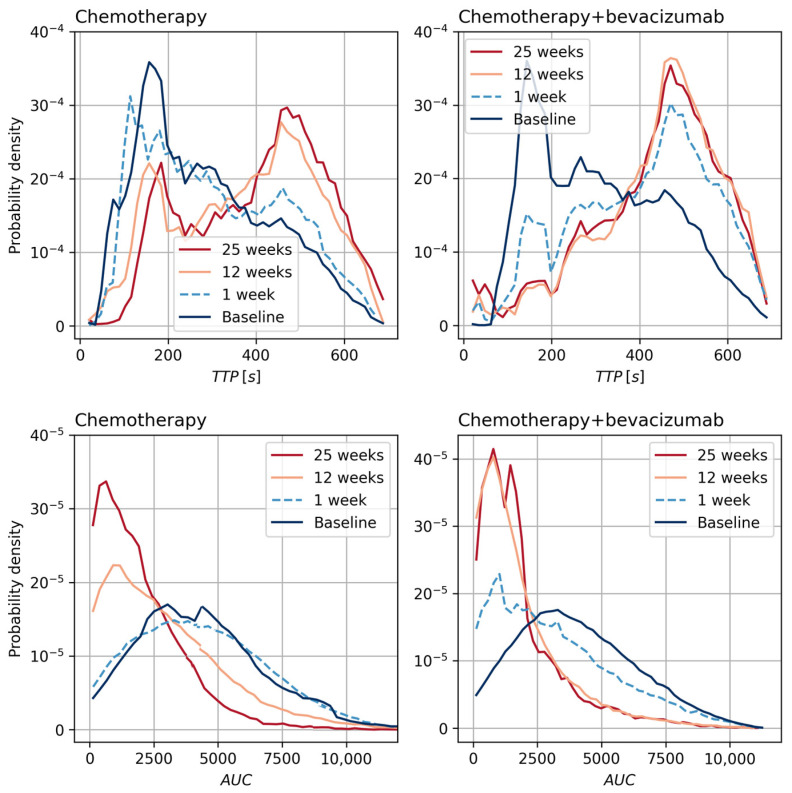
Probability density plots of time-to-peak (TTP) and area under the contrast curve (AUC) for individual breast tumor voxels during treatment with neoadjuvant chemotherapy-only (*n* = 32) or chemotherapy + bevacizumab (*n* = 38).

To obtain an overview of the effect of the two different treatments on MR perfusion parameters, all voxels from all patients were compared between the two treatment groups and displayed as density plots. The semiquantitative parameters are shown in Figure 2.

**Figure 3:**
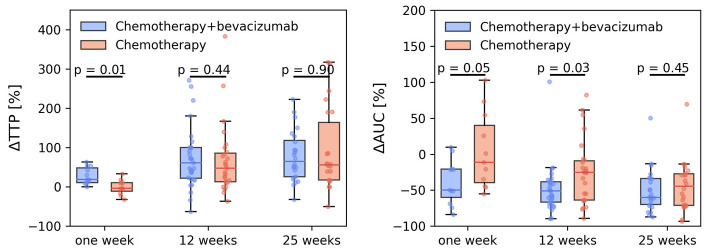
Relative change in time-to-peak (TTP) and area under the contrast curve (AUC) after 1, 12, and 25 weeks for breast cancer patients who received neoadjuvant treatment with chemotherapy-only or chemotherapy + bevacizumab. All changes are normalized to individual pretreatment values.

The distribution of TTP values for the chemotherapy-only group at one week was similar to the pretreatment distribution (Figure 2 and Figure 3). At 12 weeks, the distribution changed to two peaks: one peak with short TTP values similar to pretreatment, and one peak with substantially longer TTP values. From 12 to 25 weeks, there was no change in the TTP distribution. For the chemotherapy + bevacizumab group, the change in distribution was different. As opposed to the chemotherapy-only group, the changes occurred already at one week and almost no voxels retained the short pretreatment TTP values (Figure 2 and Figure 3). 

The distribution of AUC values for the chemotherapy-only group at one week was similar to the pretreatment distribution (Figure 2 and Figure 3). At 12 and 25 weeks there was an increasing shift towards lower AUC values. For the chemotherapy + bevacizumab group, the AUC values decreased already during the first week of treatment and the decrease continued until week 12 (Figure 2 and Figure 3). In contrast to the chemotherapy-only group, there were no further changes from 12 to 25 weeks. At 25 weeks, the distribution of AUC values for the two treatment groups was similar.

In Figure 3, changes in tumor median Δ TTP and Δ AUC are shown. In line with the voxel analysis, a significantly larger change in TTP was found for the chemotherapy + bevacizumab group at 1 week, but not for 12 and 25 weeks. The change in AUC was significantly lower in the chemotherapy + bevacizumab group at 1 week and 12 weeks. Effective treatment is reflected by longer TTP and a smaller AUC, equivalent to a transition towards a more benign-looking enhancement curve [25,26] Schnall + Kuhl.

**Figure 4:**
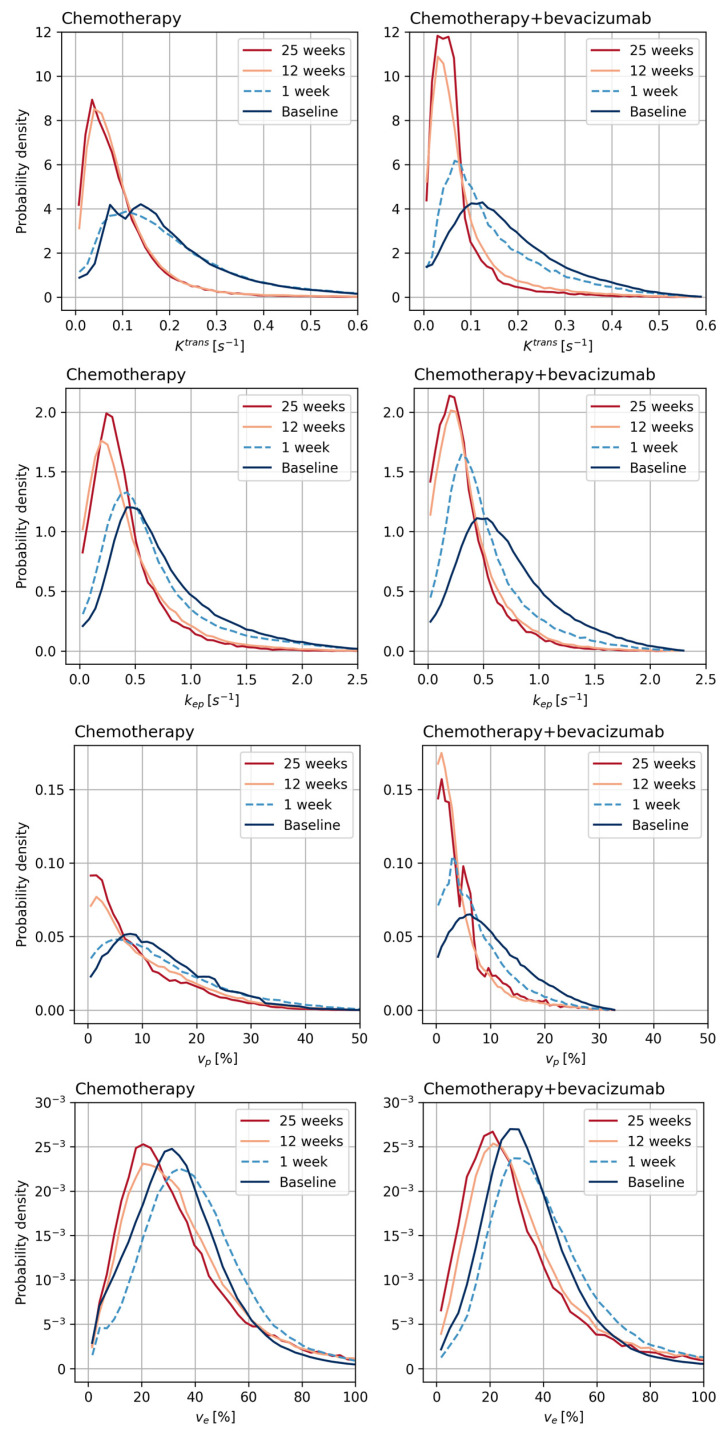
Probability density plots of $K^{trans}$, $k_{ep}$, $v_{e}$ and $v_{p}$ from the extended Tofts model for individual breast tumor voxels during treatment with neoadjuvant chemotherapy-only (control, *n* = 32) or neoadjuvant chemotherapy + bevacizumab (bevacizumab, *n* = 38).

The distributions of the parameters from the extended Tofts model in all tumor voxels in the two treatment groups during treatment are shown in the probability density plots in Figure 4.

There were no differences between the baseline values of $K^{trans}$, $k_{ep}$, $v_{e}$, and $v_{p}$ in the two treatment groups (Table 3).

**Figure 5:**
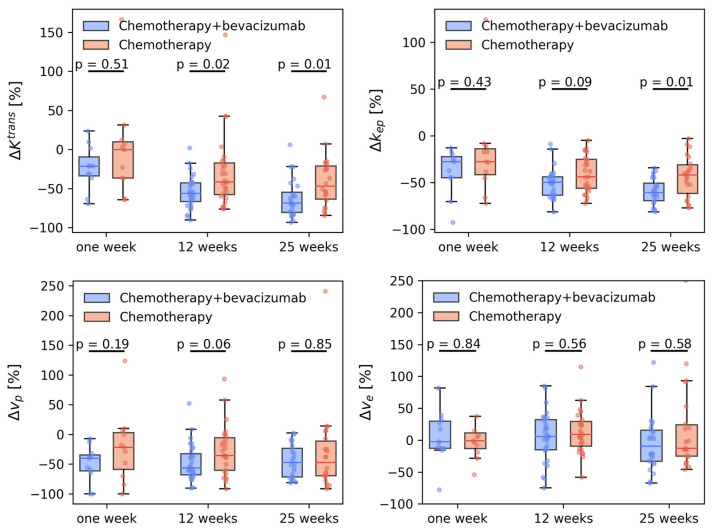
Relative change in $K^{trans}$, $k_{ep}$, $v_{e}$, and $v_{p}$ after 1, 12, and 25 weeks for the breast cancer patients who received neoadjuvant treatment with chemotherapy (controls, *n* = 32) or chemotherapy + bevacizumab (bevacizumab, *n* = 38). All changes are normalized to individual pretreatment values.

For $K^{trans}$ there was a reduction, both in the voxel-wise distributions (Figure 4) and in the median tumor values during treatment (Table 3, Figure 5). For the chemotherapy-only group, the change occurred between the first and twelfth week. For the chemotherapy + bevacizumab group, $K^{trans}$ decreased already in the first week. At 12 and 25 weeks, the reduction in $K^{trans}$ was significantly larger for the bevacizumab group (Figure 5). $k_{ep}$ showed similar development to $K^{trans}$. 

Similar to $K^{trans}$ for $v_{p}$ there was a decrease in the distribution (Figure 4) and in the median values (Table 3, Figure 5). For the chemotherapy-only group, the change occurred between the first and twelfth week. For the chemotherapy + bevacizumab group, $v_{p}$ decreased already at the first week (*p* = 2.45 × 10^−5^) without any further reduction during the next 24 weeks. The reduction in $v_{p}$ was of the same magnitude in both treatment groups at all timepoints. 

None of the patient groups showed a significant change in $v_{e}$ during the treatment.

### 3.3. Tumor Vascular Architecture

**Figure 6:**
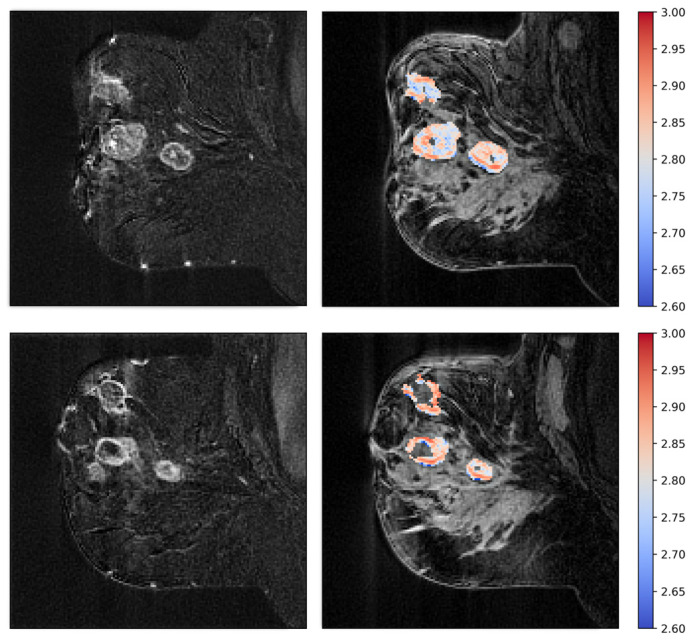
Patient treated with bevacizumab showing intermediate pre-treatment fractal dimension (**top**), with a significant increase in fractal dimensionality after one week of treatment (**bottom**). The fractal dimension maps are shown to the right as colored overlays on T2-weighted images. To the left are the subtraction images that the fractal dimension is calculated from.

**Figure 7:**
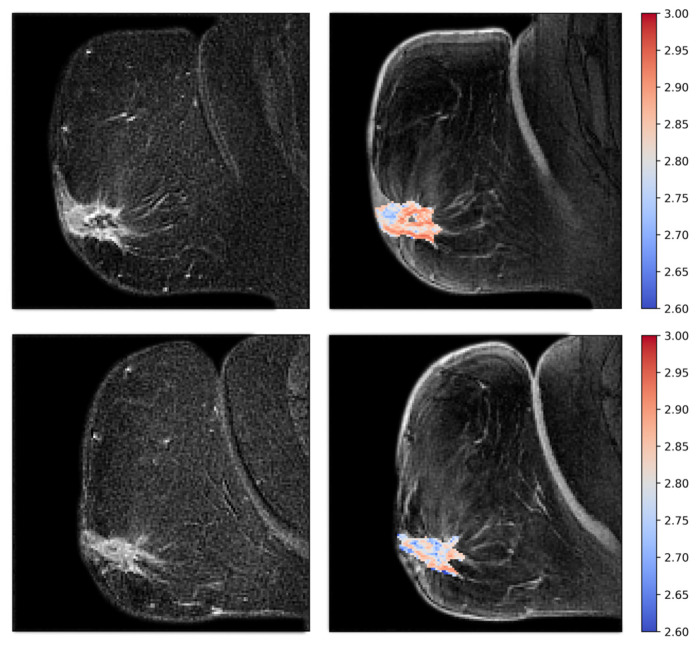
Patient treated with bevacizumab, showing high pretreatment fractal dimension (**top**), with a significant reduction after one week of treatment (**bottom**). Fractal dimension maps are shown to the right, and subtraction images are shown to the left.

Examples of fractal dimension maps for two of the patients are shown in Figure 6 and Figure 7.

**Figure 8:**
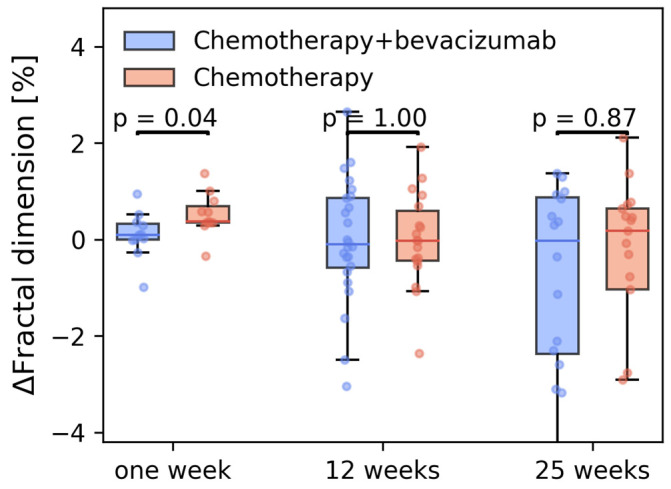
Relative change in the fractal dimensionality of the subtraction images in the breast cancer patients who received neoadjuvant treatment with chemotherapy (controls, *n* = 32) or chemotherapy + bevacizumab (bevacizumab, *n* = 38).

There were no differences in mean fractal dimensions at baseline in the two treatment groups (*p* = 0.89). Treatment led to changes in image texture over time for individual patients (Figure 8). Patients in the chemotherapy-only group had a significant and transient increase in fractal dimensionality after 1 week (*p* = 0.04) that returned to pretreatment levels within 12 weeks. This was not observed in the chemotherapy + bevacizumab group. In both groups, there was a large increase in the spread in the fractal dimension at 12 weeks, which increased further at 25 weeks. However, the mean values were not significantly different between the two groups and between these two time points. These mean/values were also not significantly different from baseline. 

There was no correlation/association between baseline tumor volumes and any of the measures of tumor functionality or architecture (Supplementary Figure S1).

## 4. Discussion

In this study, we wanted to investigate the anticipated anti-angiogenic effects of bevacizumab in patients with breast cancer. We used vascular parameters derived from clinical DCE-MRI and compared the results from two groups of patients, one treated with chemotherapy only and one with bevacizumab added to the chemotherapy. We recorded data at baseline and at 1, 12, and 25 weeks, and we observed changes during treatment for all semi-quantitative and quantitative parameters. Importantly, patients treated with chemotherapy and Bevacizumab saw a more rapid change in the semi-quantitative parameters representing vascular shutdown, with changes occurring already at week 1. In contrast, for the patients treated with chemotherapy alone, similar changes were observed at the week 12 timepoint. 

There are several preclinical but few clinical studies in solid cancers reporting on changes in DCE parameters during bevacizumab treatment alone [27,28] or in combination with cytostatic treatment [29]. The preclinical results are conflicting; both increases and decreases in DCE-MRI parameters have been reported [30,31]. The two clinical studies reported a decrease in DCE-MRI parameters. Willet et al. found vascular shutdown at two weeks in rectal cancer patients using dynamic computed tomography (CT) [27] López-Vega et al. reported a decrease in all DCE parameters at 12 weeks [29]. Our finding of vascular shutdown at one week in the bevacizumab + chemotherapy group is in accordance with the findings of Willet et al. in rectal cancer. At 12 weeks we found significantly decreased DCE parameters for all patients, in accordance with López-Vega et al. 

In a recent review article, Magnussen and Mills describe vascular normalization of the tumor microenvironment following anti-angiogenic treatment. Normal vasculature depends on a balance between pro-angiogenetic and anti-angiogenetic factors. The tumor microenvironment disturbs this balance and promotes sprouting angiogenesis and other forms of vessel formation [32] which leads to abnormal vasculature with abnormal blood perfusion. Pro-angiogenetic imbalance also causes irregular and tortuous vascular architecture, resulting in high fractal dimension on DCE-MRI images [10,23]. Anti-angiogenic treatment can re-establish the balance and restructure the vascular architecture [9]. In the following, based on the observed changes in the DCE-MRI, we will try to interpret and explain the changes in the vasculature based on the concept of sprouting angiogenesis, and its effects on blood flow and vessel permeability. 

Tumor perfusion in the two groups was significantly different already at one week. In the bevacizumab group, the TTP increased in almost all tumor voxels, whereas in the chemotherapy-only group, the increase in TTP occurred later and did not involve all tumor voxels. Compared to TTP, the treatment effect seen on AUC is similar but slower and incremental. The impact on AUC is more dependent on the amount of the contrast enhancement as compared to TTP which is more impacted by the slope of the contrast enhancement curve. Therefore, we hypothesize that bevacizumab shuts down leaky, tortuous, abnormal vessels faster than smaller, well-differentiated, well-functioning vessels. This would be in line with Magnussen and Mills who describe that anti-angiogenic treatment mainly blocks the sprouting angiogenesis [9]. $K^{trans}$ was significantly reduced after one week only in the Bevacizumab group, yet the difference in the reduction between the treatment groups was less pronounced than in the semiquantitative parameters. $K^{trans}$ is usually interpreted as a measure of vascular permeability, and as sprouting angiogenesis tends to result in highly permeable vessels, it is reasonable to interpret a reduction in $K^{trans}$ as in accordance with a normalization of the tumor vasculature resulting from a reduction in sprouting angiogenesis. When interpreting $K^{trans}$ as a measure of vascular permeability, however, we implicitly assume either negligible blood volume or very high blood flow. Neither of these conditions can be safely assumed in highly tortuous cases with $v_{p}$ in the order of 10%. Studies have shown that in cases of intermediate vascularization ($v_{p}$~10%) and intermediate blood flow, $K^{trans}$ is strongly correlated to the blood flow [18]. In light of this, the changes in $K^{trans}$ are consistent with the changes in TTP and AUC. The rapid change in $K^{trans}$ in the bevacizumab group indicates that there is an abrupt reduction in inflow. Because this effect was observed before any shrinkage of the tumor had occurred, this suggests that the initial mechanism of bevacizumab is a rapid shutdown of tumor vascularization. Tumor architecture was also significantly different between the two treatment groups at one week. The fractal dimension increased in the chemotherapy-only group, but not in the bevacizumab group. Given that fractal dimensionality is linked to tortuosity of tumor vessels, this was surprising. A possible explanation could be that chemotherapy induced a flare effect of tumor vascularization that was prevented by adding bevacizumab. The fractal analyses were performed on late DCE-MRI images (238 s) ensuring maximal signal-to-noise ratio (SNR). Performing the fractal analyses on early DCE images more selectively could have reflected the fractal dimension of the tumor vessel architecture but at the expense of the SNR. This method needs further investigation and standardization. At 12 and 25 weeks, there was no significant change in the mean fractal dimension compared to baseline. There are two possible explanations for the absence of measurable change. Either there is no change, or it cannot be measured because the volume, i.e., the number of voxels, is too small. Furthermore, at these late timepoints a large therapeutic effect has occurred and, although tumor volume was limited to areas of contrast enhancement at all timepoints, the VOI may contain an admixture of residual tumor islets and benign reactive tissue. 

Tumor volume did not change at one week but was significantly reduced at 12 and 25 weeks without difference between the treatment groups. Some tumors showed a rather large percentual increase in the first week. We think there are two likely explanations: First, in small and fragmented tumors, minimal changes in the segmentation might lead to a large percentual increase. Second, at these early time points peritumoral inflammation, appearing similar to tumor at DCE, might have been included in the segmentation. The volume reduction in the chemotherapy-only group was so large (89%) that any additional volume reduction resulting from adding bevacizumab would be difficult to detect.

Our study has several limitations. Only one-third of the patients were examined at one week, thus we have significantly lower statistical power at this early time point. We have not evaluated the mechanism of bevacizumab alone but only in combination with chemotherapy. Without a reference tissue, the hypothesis of vascular normalization is difficult to confirm; however, representative breast tissue is impracticable to define. A further limitation is that the interpretation of the pharmacokinetic models is challenging. Our 13 s temporal resolution of the DCE-MRI is higher than the standard clinical routine, but not optimal for the extended Tofts model. Accurate perfusion parameter estimates are also influenced by the sampling of the AIF. A reproducible AIF is difficult to obtain in the breast because the main feeding arteries are small. Thus, the AIF must be sampled from within the heart. An AIF from the heart is subject to dispersion or delay that occurs as the contract agent travels through the tissue from the ventricle to the breast [16]. Ideally, to better understand the mechanism of vascular changes, the patients could have been examined at more time points, especially in the early phase of treatment, but in clinical studies there are limits to what can be achieved. 

Methodologically, the study was conducted according to the recommendations for appropriate methodology for clinical trials for the assessment of anti-angiogenic and anti-vascular therapeutics using MRI [14,15]. Furthermore, we studied the mechanism in vivo using standard clinical methods that can be used in clinical routine and clinical studies to measure early response to anti-angiogenic treatment. Although anti-angiogenic treatment has failed to show improved overall survival, it has been reported to improve progression-free survival, suggesting that there might be a subgroup of patients that benefit. Using protein signature and DNA methylation data, Haugen et al. identified subgroups of breast cancer patients with pathological complete response [7]. In this study, we have shown that DCE-MRI demonstrates vascular shutdown DCE-MRI might be a clinical tool to help identify patients that benefit from anti-angiogenic treatment. We are currently conducting a prospective study combining protein and gene analyses with DCE to select patients and assess treatment response in breast cancer patients (EudraCT Number: 2021-005850-27).

## 5. Conclusions

In this clinical study of 70 locally advanced breast cancer patients, adding bevacizumab to standard chemotherapy led to rapid changes in DCE-MRI parameters at one week, reflecting vascular shutdown. At one week, the fractal dimension of the chemotherapy-only group was significantly higher. At 12 and 25 weeks, there were no differences between the two treatment groups.

## Figures and Tables

**Table 1:** Baseline patient and tumor characteristics.

Characteristic	All	Chemotherapy	Chemotherapy+ Bevacizumab	*p*-Value
Patients (*N*)	70	32	38	
Age (years)				0.86 (*t*-test)
Mean	49.3	49.0	49.4	
Median	49.5	50.5	48.0	
Range	30–70	30–64	31–70	
Tumor stage				0.42 (ANOVA)
T2	20	10	10	
T3	46	20	26	
T4	4	2	2	
Tumor size [mm]				0.70 (*t*-test)
Mean	46.8	46.1	47.5	
Range	16–92	16–92	29–76	
Lymph node status ^1^				
cN0	36	15	21	0.79 (ANOVA)
cN1	5	4	1	
pN1	29	13	16	
Type				
Ductal carcinoma	55	26	30	1.00 (Fischer’s exact test)
Lobular carcinoma	14	6	8	
Grade				
1	6	0	6	0.41 (ANOVA)
2	49	24	25	
3	14	7	7	
N/A	1	1	0	
Estrogen receptor status				
Positive	59	27	32	1.00 (Fischer’s exact test)
Negative	11	5	6	

Abbreviations: N/A = not available, ANOVA = analysis of variance. ^1^ cN: palpable malignant nodes, not verified by fine needle aspiration; pN: malignant cells in nodes verified by fine needle aspiration.

**Table 2:** Semi-automatically segmented tumor volumes (median, interquartile range) before and after 1, 12, and 25 weeks for the breast cancer patients who received neoadjuvant treatment with chemotherapy alone or chemotherapy + bevacizumab (bevacizumab. Change in tumor volumes ΔVolume is percent change relative to individual pretreatment volumes.

	N	$\mathrm{Volume}\left[{\mathrm{cm}}^{3}\right]$	Δ Volume [%]
	Chemotherapy		
Baseline	32	9.7 (5.8–21.6)	
1 week	13	14.4 (6.6–24.0)	19.2 (−7.0–24.6)
12 weeks	31	3.8 (2.1–6.5)	−66 (−89.0–23.6)
25 weeks	29	0.9 (0.2–2.5)	−88.5 (−98.6–(−74.7))
	Chemotherapy + bevacizumab		
Baseline	38	11.1 (8.5–15.5)	
1 week	13	11.4 (8.0–15.1)	+4.4 (−16.6–27.3)
12 weeks	35	1.9 (1.0–7.1)	−77.8 (−90.8–(−47.7))
25 weeks	35	1.0 (0.2–3.2)	−89.8 (−96.7–(−68.4))
	All		
Baseline	70	10.4 (6.1–18.7)	
1 week	26	11.9 (6.6–21.6)	14 (−6.1–27.1)
12 weeks	66	2.9 (1.4–7.1)	−73.7 (−90.8–(−41.8)
25 weeks	64	1.0 (0.2–2.9)	−89.8 (−98.3–(−70.2))

**Table 3:** $K^{trans}$, $k_{ep}$, $v_{e}$, and $v_{p}$ from the extended Tofts model at baseline, and after 1, 12, and 25 weeks for the breast cancer patients who received neoadjuvant treatment with chemotherapy (controls, *n* = 32) or chemotherapy + bevacizumab (bevacizumab, *n* = 38). The median and the 95% confidence interval of the four parameters and their relative changes from baseline are shown.

	$K^{trans}$	$k_{ep}$	$v_{p}$	$v_{e}$
	Chemotherapy			
Baseline	0.13 (0.11, 0.15)	0.55 (0.48, 0.61)	9.4 (8.11, 10.69)	23.92 (20.98, 26.86)
1 week	0.11 (0.08, 0.14)	0.36 (0.27, 0.45)	8.41 (2.14, 14.67)	24.97 (19.99, 29.94)
12 weeks	0.08 (0.06, 0.11)	0.31 (0.25, 0.37)	6.25 (4.48, 8.02)	30.6 (18.81, 42.39)
25 weeks	0.07 (0.05, 0.09)	0.3 (0.24, 0.35)	6.61 (3.2, 10.02)	24.61 (20.25, 28.98)
Δ1 week [%]	−0.3 (−42.4, 41.9)	−18.7 (−53.6, −33.8)	−21.0 (−61.1, 19.1)	26.0 (−43.7, 95.7)
Δ12 weeks [%]	−31.4 (−50.6, −12.2)	−41.2 (−49.5, −32.9)	−28.9 (−46.9, −10.8)	40.0 (−18.3, 98.3)
Δ25 weeks [%]	−39.4 (−55.5, −23.3)	−43.7 (−53.6, −33.8)	−28.7 (−59.2, 1.8)	18.8 (−13.1, 50.7)
	Chemotherapy + bevacizumab			
Baseline	0.14 (0.12, 0.16)	0.55 (0.5, 0.6)	7.83 (6.78, 8.87)	25.98 (23.94, 28.02)
1 week	0.08 (0.05, 0.11)	0.27 (0.16, 0.37)	2.8 (1.38, 4.21)	22.9 (14.22, 31.57)
12 weeks	0.06 (0.05, 0.07)	0.27 (0.24, 0.3)	3.8 (2.99, 4.61)	26.12 (22.35, 29.9)
25 weeks	0.05 (0.04, 0.06)	0.22 (0.19, 0.25)	3.39 (2.71, 4.07)	24.33 (20.34, 28.33)
Δ1 week [%]	−29.8 (−52.6, −7.0)	−44.4 (−64.6, −24.2)	−53.7 (−76.3, −31.1)	−6.6 (−39.8, 26.5)
Δ12 weeks [%]	−54.3 (−61.8, −46.8)	−50.7 (−56.4, −45.0)	−47.2 (−58.4, −36.0)	6.1 (−7.9, 20.0)
Δ25 weeks [%]	−60.5 (−71.1, −49.9)	−58.9 (−64.5, −53.2)	−46.0 (−57.4, −34.6)	−1.5 (−20.1, 17.2)

## Data Availability

The imaging datasets generated and analyzed during the current study are available from the corresponding author upon request.

## Supplementary Materials

The following supporting information can be downloaded at: https://www.mdpi.com/article/10.3390/cancers15184662/s1, Figure S1: Pre-treatment correlations between the MRI parameters and tumor volume; Table S1: MRI protocol of the NeoAva study.
