# Supplementary material for: MRI Assessment of Changes in Tumor Vascularization during Neoadjuvant Anti-Angiogenic Treatment in Locally Advanced Breast Cancer Patients
Source: Cancers (Basel). 2023 Sep 21;15(18):4662. doi: 10.3390/cancers15184662 (PMC10526130; doi:10.3390/cancers15184662)
Supplement: Supplementary file 1 [file cancers-15-04662-s001.zip › cancers-2565843-supplementary.pdf]

Supplementary figures

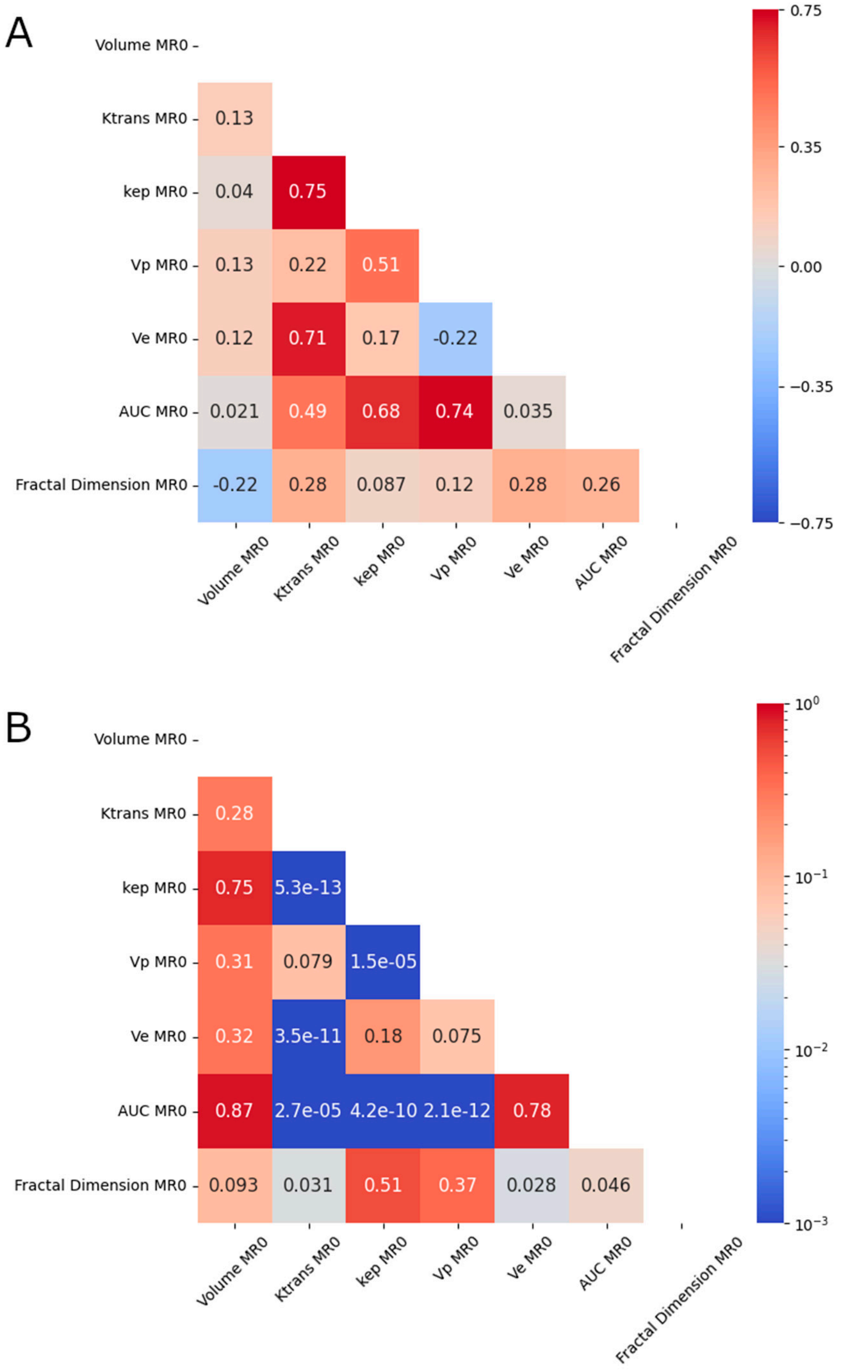

Supplementary Figure S1: *Pre-treatment correlations between the MRI parameters and tumor volume. Pearson correlation coefficients are shown at the top (A), and p-values are shown at the bottom (B).*

Supplementary table

Supplementary Table S1: *MRI protocol of the NeoAva study.*

**Acquisition parameters**	**TSE T2W**	**TSE T1W**	**T1W GRE***	**3D SPGR DCE**	**DW SE single-shot EPI**
Plane	Transversal	Sagittal	Tranversal	Transversal	Transversal
Echo time [ms]	80	9.4	1.21	2.59	69
Repetition time [ms]	4570	666	4.0	5.46	5200
Field of view [mm]	340 x 340	220 x 220	320 x 320	320 x 320	360 x 185
Pixel size [mm]	0.66 x 0.66	0.86 x 0.86	1.0 x 1.0	1.0 x 1.0	2.57 x 2.57
Slice thickness [mm]	3	4	1.5	1.5	4.5
Number of exitations	1	1	1	1	6
Temporal resolution [s]				13	
b-values [s/mm^2]					0, 50, 250, 500, 800
Echo train length	17	5			
Bandwith [Hz/px]	222	219		460	1116

TSE = turbo spin echo, GRE = gradient echo, SPGR = spoiled gradient echo, DCE = dynamic contrast-enhanced, DW = diffusion weighted, SE = spin echo, EPI = echo planar imaging

**Performed twice for corection of B1 inhomogeniteies, once using only the bodycoil, and once using both the bodycoil and the breast-coil*
